# Surgical frailty assessment: a missed opportunity

**DOI:** 10.1186/s12871-017-0390-7

**Published:** 2017-07-24

**Authors:** Gilgamesh Eamer, Jennifer A. Gibson, Chelsia Gillis, Amy T. Hsu, Marian Krawczyk, Emily MacDonald, Reid Whitlock, Rachel G. Khadaroo

**Affiliations:** 1 Department of Surgery, 2D, Walter C Mackenzie Health Sciences Centre, University of Alberta, 8440-112 Street, Edmonton, AB T6G 2B7 Canada; 2 School of Public Health, University of Alberta, Edmonton, AB Canada; 3 School of Nursing, University of British Columbia, Vancouver, BC Canada; 4 Cumming School of Medicine, University of Calgary, Calgary, AB Canada; 5 Clinical Epidemiology Program, Ottawa Hospital Research Institute, Ottawa, ON Canada; 6 Department of Epidemiology and Community Medicine, University of Ottawa, Ottawa, ON Canada; 7 Centre for Health Evaluation and Outcome Sciences, St. Paul Hospital, Vancouver, BC Canada; 8 Trinity Western University, Langley, BC Canada; 9 Department of Nursing and Health Sciences, University of New Brunswick, Saint John, NB Canada; 10 Department of Community Health Sciences, University of Manitoba, Winnipeg, MB Canada

**Keywords:** Frailty, Operative screening, Improving care, Survey, Surgery

## Abstract

**Background:**

Preoperative frailty predicts adverse postoperative outcomes. Despite the advantages of incorporating frailty assessment into surgical settings, there is limited research on surgical healthcare professionals’ use of frailty assessment for perioperative care.

**Methods:**

Healthcare professionals caring for patients enrolled at a Canadian teaching hospital were surveyed to assess their perceptions of frailty, as well as attitudes towards and practices for frail patients. The survey contained open-ended and 5-point Likert scale questions. Responses were compared across professions using independent sample t-tests and correlations between survey items were analyzed.

**Results:**

Nurses and allied health professionals were more likely than surgeons to think frailty should play a role in planning a patient’s care (nurses vs. surgeons *p* = 0.008, allied health vs. surgeons *p* = 0.014). Very few respondents (17.5%) reported that they ‘always used’ a frailty assessment tool. Results from qualitative data analysis identified four main barriers to frailty assessment: institutional, healthcare system, professional knowledge, and patient/family barriers.

**Conclusion:**

Across all disciplines, the lack of knowledge about frailty issues was a prominent barrier to the use of frailty assessments in practice, despite clinicians’ understanding that frailty affects their patients’ outcomes. Confidence in frailty assessment tool use through education and addressing barriers to implementation may increase use and improve patient care. Healthcare professionals agree that frailty assessments should play a role in perioperative care. However, few perform them in practice. Lack of knowledge about frailty is a key barrier in the use of frailty assessments and the majority of respondents agreed that they would benefit from further training.

**Electronic supplementary material:**

The online version of this article (doi:10.1186/s12871-017-0390-7) contains supplementary material, which is available to authorized users.

## Background

Frailty is a multisystem syndrome of low physiological reserves, coupled with a diminished capacity to respond to stressors, resulting in an increased risk of adverse events [1] and has been identified as a useful concept for care [2]. There is a growing body of evidence that frailty is associated with an increased risk of adverse health outcomes – including mortality, loss of activities of daily living, and hospitalization [3] – particularly in surgical settings [4–7].

Surgical interventions with patients aged 65 years and older have become increasingly common [8]. While adverse postoperative events and prolonged hospital length of stay are more common in older people [9–14], age is not the primary risk factor for poor outcomes. Preoperative frailty is more important than age as a predictor of poor postoperative outcomes [9, 15]. The value of identifying frailty as an important risk factor for adverse health outcomes among hospitalized older adults is well-established. Multiple frailty assessment tools have been developed and used in hospital settings; including Frailty Phenotype [16], falls, the Edmonton Frail Scale [17] and the Clinical Frailty Scale (CFS) [1]. These types of frailty screening and the implementation of early interventions has been associated with a preserved autonomy and reduced occurrence of adverse health events [18–20]. Comparatively, the CFS is less cumbersome than the Frailty Phenotype, and has demonstrated very good inter-rater reliability. The CFS has been found to be an independent predictor of inpatient, 18-month and 70-month mortality, hospital length of stay, and falls [1, 21–23].

Despite strong associations between frailty and poor health outcomes, the assessment of frailty in older adults is not routine surgical practice [24]. Furthermore, little is known about interdisciplinary surgical Healthcare Professionals’ (HCP) perception of frailty, or its clinical assessment. The aim of this study was to explore interdisciplinary hospital HCPs’ self-reported beliefs regarding: 1) the importance of formally assessing frailty, 2) the usefulness of a specific frailty scale score, 3) barriers to using frailty assessments in clinical practice, and 4) to establish if there are any significant differences in perspectives between health care disciplines.

## Methods

### Study population

The Elder-friendly Approaches to the Surgical Environment (EASE) project is ongoing at two teaching hospitals in Alberta, Canada (clinicaltrials.gov registration, NCT02233153). The broad aim of the EASE project was to investigate the use of comprehensive geriatric assessment (CGA) for post-operative emergency abdominal surgery patients [25]. As a component of the CGA, all enrolled patients are screened with the CFS and given a frailty score. All interdisciplinary HCPs working on the EASE surgical units were offered education during an in-service session about frailty, importance of frailty assessment, and best practices for older adults.

### Survey development

As no validated survey exists, we developed a survey to capture the HCPs’ views on frailty and its relevance to care planning. Content validity was maintained by incorporating expert consultation and input from our interdisciplinary team and surgical staff. Survey items were then rated and organized into a survey. A cognitive interviewing approach was used for pilot testing with two physicians, two RNs and two allied health professionals following initial survey development. This was done to assess the survey’s construct validity, interpretability, redundancy and ease of administration. Based on feedback received, the initial survey was modified to improve the interpretation of the questions and reduce response burden. The subjects’ responses were recorded and evaluated within our interdisciplinary team [26] then revised and piloted with four additional HCPs until revisions were no longer necessary. A clinical sensibility-testing tool [27] ensured validity. Survey reliability was assessed post hoc using Cronbach’s alpha for Likert-scale and yes or no questions. Cronbach’s alpha for the overall survey and two main themes were measured. The main themes were (1) frailty assessment of patients (Pages 2 and 3 of Additional file 1) and (2) awareness of the EASE study and Clinical Frailty Scale used in the EASE study (pages 4 and 5 of Additional file 1). Cronbach’s alpha values were pre-defined as acceptable if it was 0.7 or higher, questionable if it was between 0.6 and 0.7 and unacceptable if it was below 0.6. The final survey contains four demographic questions, fourteen five-point Likert scale questions, ranging from ‘1′ (strongly disagree) to ‘5′ (strongly agree), and a “not applicable/no comment” option, four yes or no questions and four open-ended questions (Additional file 1).

### Data collection

Data collection occurred between October and November 2016. Electronic or paper-based surveys were distributed to all interdisciplinary HCPs working within the EASE project clinical areas. Participation was voluntary and confidential. The survey was attached to a cover letter inviting participants to complete the survey and included contact information and proof of ethic approval (Additional file 1). Consent was implied by voluntary and anonymous completion of the survey. Ethical approval was obtained at the University of Alberta (Protocol no. Pro00064524).

### Analysis

For the quantitative analysis, HCPs were clustered into three subgroups: nurses (Registered Nurses [RNs] and Licensed Practical Nurses [LPNs]), surgeons (surgeons and surgical residents), and allied health professionals (dietitians, occupational therapists, physiotherapists, social workers, and service aid). Mean response scores, frequencies and percent “agree” or “strongly agree” for overall group and subgroups were calculated for each survey item using available case analysis. To determine whether there were significant differences in the mean response scores between pairs of HCP subgroups, we conducted t-tests for independent samples. We also used analysis-of-variance (ANOVA) models to compare the mean response scores across all HCP subgroups, collectively, in a sensitivity analysis. Spearman’s rank-order correlations were computed to measure the strength and direction of association between pairs of ranked, categorical variables.

The free-text survey components were analyzed using qualitative thematic analysis. Responses were also examined according to the three disciplinary subgroups.

## Results

### Overall

A total of 117 HCPs (17 surgeons, 30 residents, 53 nurses, 5 occupational therapists, 4 dietitians, 4 physiotherapists, 2 social workers, 2 service aids) were invited to participate by completing the research survey. Forty-nine surveys were returned, representing a 41.8% response rate. The demographic characteristics of respondents are presented in Table 1. The final study sample consisted of 20 surgeons (14 residents, 6 surgeons), 16 nurses (13 RNs, 3 LPNs), and 13 allied health professionals (4 dietitians, 3 occupational therapists, 3 physiotherapists, 2 social workers, 1 service aid). Respondents were predominantly female (67.3%) and over half (59.2%) were between the ages of 25 and 34. Mean years of experience in surgery was 11.4 years (*SD* = 8.1). Surgeons and resident surgeons in training were pooled together. To ensure that the additional clinical experience of the surgeons wasn’t influencing their practice patterns with respect to frailty we conducted t-tests for independent samples between the surgeons and resident surgeons and found there was no statistically significant difference between the responses from staff surgeons and surgical residents. Cronbach’s alpha assessment of survey reliability was ‘acceptable’ for the overall study (α=0.79), ‘acceptable’ for the frailty assessment theme (α=0.70) and questionable for the EASE/CFS theme (α=0.68).

**Table 1:** Demographic profile of survey respondents

	Nurses		Surgeons		Allied health		Total	
Number of respondents	16		20		13		49	
		(percent of subgroup)		(percent of subgroup)		(percent of subgroup)		(% of respondents)
Age								
Under 25	2	(12.5%)	0	(0.0%)	0	(0.0%)	2	(4.1%)
25–34	9	(56.3%)	15	(75.0%)	5	(38.5%)	29	(59.2%)
35–44	0	(0.0%)	1	(5.0%)	3	(23.1%)	4	(8.2%)
45–54	2	(12.5%)	3	(15.0%)	1	(7.7%)	6	(12.2%)
55 and over	3	(18.8%)	1	(5.0%)	4	(20.0%)	8	(16.3%)
Gender								
Male	3	(18.8%)	10	(50.0%)	1	(7.7%)	14	(28.6%)
Female	12	(75.0%)	9	(45.0%)	12	(92.3%)	33	(67.3%)
Prefer not to disclose	1	(6.3%)	1	(5.0%)	0	(0.0%)	2	(4.1%)
Experience								
Years in profession, mean ± SD	20.6	± 13.1	7.8	± 8.4	13.8	± 9.5	13.5	± 10.6
Years in surgery, mean ± SD	17.7	± 9.6	7.6	± 7.5	9.5	± 7.4	11.4	± 8.2
Years on current unit, mean ± SD	12.9	± 5.6	7.0	± 7.8	7.8	± 6.9	9.1	± 6.7

### Survey results

Seven survey questions captured interdisciplinary HCPs’ perceptions of the importance of frailty assessment scores in clinical practice (Table 2). Most HCPs supported 5 of the 7 statements including strong agreement that the frailty of a patient should play a role in planning patients’ perioperative hospital care. However, only 7 (17.5%) of the HCPs always used a frailty assessment tool to assess patients. Furthermore, only 17 (38.6%) of respondents were confident in their ability to assess frailty. Confidence in ability to assess frailty was positively correlated with belief that frailty assessment is important for care planning (*r* ^*s*^ = 0.331, *p* = 0.028) and delivering perioperative care (*r* ^*s*^ = 0.450, *p* = 0.003).

**Table 2:** Perceived importance of frailty assessment across disciplines/professions

	Nurses	Surgeons	Allied health	Total
Number of respondents	16	20	13	49
A frailty assessment should be done for all surgical patients	9/13 (69.2%) 3.7	11/20 (55.0%) 3.4	10/13 (76.7%) 3.9	30/46 (65.2%) 3.6
It is part of my professional role/responsibility to assess patients for frailty	11/13 (84.6%) 4.2	14/20 (70.0%) 3.8	7/12 (58.3%) 3.3	32/45 (71.1%) 3.7
I always use a frailty assessment tool to assess patients for frailty	3/12 (25.0%) 2.5	3/20 (15.0%) 2.2	1/8 (12.5%) 2.5	7/40 (17.5%) 2.3
I am confident in my ability to assess patients for frailty	7/13 (53.8%) 3.2	5/20 (25.0%) 2.8	5/11 (45.5%) 2.8	17/44 (38.6%) 2.9
The frailty of a patient should play a role in planning a patient’s perioperative care in the hospital	16/16 (100%) 4.6	20/20 (100%) 4.2	13/13 (100%) 4.6	49/49 (100%) 4.4
The frailty of a patient always plays a role in my planning of a patient’s perioperative care in the hospital	14/16 (87.5%) 4.0	10/20 (50.0%) 3.4	11/13 (84.6%) 4.4	35/49 (71.4%) 3.9
Frailty is an important factor in how I provide a patient’s perioperative care in the hospital	14/15 (93.3%) 4.3	13/20 (65.0%) 3.7	11/11 (100%) 4.5	38/46 (82.6%) 4.1

Not all respondents answered all questions. Each item above represents all people who strongly agreed or agreed (numerator) divided by the number of responses to that question (denominator). The mean score (1 = “Strongly disagree”, 3 = “Neither agree nor disagree”, and 5 = “Strongly agree”) is reported below

Although all subgroups supported “The frailty of a patient should play a role in planning a patient’s perioperative care in the hospital”, nurses and allied health professionals were more likely to report that they “strongly agree” with this statement in comparison to surgeons (nurses vs. surgeons: 4.6 vs. 4.2; *p* = 0.008, allied health vs. surgeons: 4.6 vs. 4.2; *p* = 0.014). Compared to the surgeon subgroup, more allied health respondents agreed (allied health vs. surgeons: 4.4 vs. 3.4) that “the frailty of a patient always plays a role in my planning of a patient’s perioperative care in the hospital” (*p* = 0.002). Additionally, nurses and allied health respondents had significantly stronger endorsement of “frailty is an important factor in how I provide a patient’s perioperative care in the hospital” (nurses vs. surgeons: 4.3 vs. 3.7; *p* = 0.008, allied health vs. surgeons: 4.5 vs. 3.7; *p* = 0.001). There was no statistical difference between nursing and allied health respondents. As expected, there was an overall positive correlation (*r* ^*s*^ = 0.228, *p* = 0.128) between the importance of identifying patient frailty (i.e., belief that a frailty assessment should be done for all surgical patients) and the HCP’s incorporation of frailty in their own care planning; with the strongest correlation among allied health (*r* ^*s*^ = 0.300, *p* = 0.319), followed by surgeons (*r* ^*s*^ = 0.242, *p* = 0.305) and nurses (*r* ^*s*^ = 0.022, *p* = 0.942); the correlations were not statistically significant for any of the HCP subgroups.

Four survey questions (3 Likert scale questions (Table 3) and one open ended question) captured interdisciplinary HCPs’ perceptions of the usefulness of frailty assessment scores in clinical practices. Despite the formalized roll-out of frailty education as a component of the EASE project, the majority of respondents (59%) answered that they were not aware of the CFS. Less than half of respondents (46.2%) in all disciplines agreed that the CFS score is useful to perioperative care in the hospital. However, awareness of the CFS was not significantly correlated with belief that it is useful to the overall perioperative care that is provided in the hospital (*r* ^*s*^ = 0.310, *p* = 0.124).

**Table 3:** Perceived usefulness of the CFS score across disciplines/professions

	Nurses	Surgeons	Allied health	Total
Number of respondents	16	20	13	49
The CFS score is useful to the overall perioperative care that is provided in the hospital	4/8 (50.0%) 3.6	5/11 (45.5%) 3.5	3/7 (42.9%) 3.4	12/26 (46.2%) 3.5
The CFS Score is useful to the perioperative care that I provide in the hospital	4/8 (50.0%) 3.5	3/11 (27.3%) 3.1	2/7 (28.6%) 3.3	9/26 (34.6%) 3.3
I would like to use or continue using the CFS score in my care of older adults	7/11 (63.6%) 3.8	7/13 (53.8%) 3.5	5/7 (71.4%) 3.7	19/31 (61.3%) 3.7

Not all respondents answered all questions. Each item above represents all people who strongly agreed or agreed (numerator) divided by the number of responses to that question (denominator). The mean score (1 = “Strongly disagree”, 3 = “Neither agree nor disagree”, and 5 = “Strongly agree”) is reported below

There were no statistical differences in the survey scores among HCPs regarding the usefulness of the CFS score. This was supported by our qualitative data; of those who chose to expand on the usefulness of the CFS score to their discipline, half indicated that it was useful.

### Barriers to frailty assessment

Respondents in all three subgroups (58.7% overall) supported “I face barriers to providing in-hospital care for patients who are frail”. Twenty-two respondents provided further detail, which we organized into four key categories: hospital-specific institutional, health care system, professional knowledge, and patient/family members.

All disciplines emphasized the workload and staffing shortages as significant hospital-specific institutional barriers. Nurses and allied health professionals described the lack of equipment as a barrier, while allied health also reported a lack of space as an obstacle. Allied health respondents identified medical team communication challenges as a barrier. Both allied health and surgeons reported unique challenges generated by the surgical setting. Surgeons focused on increased post-surgical care requirements and subsequent discharge challenges in caring for patients who are frail. One dietitian clearly articulated the tension that can accrue between professional goals in caring for pre-operative patients, where “[we] work hard to optimize intake before surgery but patients are often fasting for procedures and have many interruptions to their diets”. Lack of physician support regarding pre-operative nutrition concerns and limited pre-operative physiotherapy were also listed by dietitians as barriers.

Surgeons primarily cited broader health system barriers of lack of sub-acute, long-term care, and supportive services in caring for patients who are frail. Nurses identified early patient discharge requirements as a larger health system barrier. No allied health identified broader health system barriers.

Interestingly, while all disciplines identified lack of knowledge about frailty issues as a barrier, the issues emphasized varied across the disciplines; surgeons identified only self-knowledge, nurses identified lack of self-knowledge and staff knowledge, and allied health only identified staff knowledge as barriers.

Finally, barriers to providing care for frail patients in relation to communications with patient and family members differed by discipline. Surgeons predominantly highlighted issues pertaining to family members’ lack of knowledge about the association between surgical outcomes and patient frailty and subsequent challenges planning overall care and discharge goals. This perspective is evidenced in one surgeon’s response, where “patient and family understanding is often a barrier - not realizing that frailty is a predictor for patient’s outcomes influences the choices they make regarding goals of care planning, discharges, safety, etc.” Nurses expressed concerns surrounding the lack of family involvement and patient symptom burden limiting their retention of medical information. Allied health reported patient compliance as an issue.

### Additional frailty assessment training

Despite in-service sessions for nurses and physicians focused on the EASE study, the importance of frailty assessment and a brief introduction to the CFS, many staff reported lack of knowledge about frailty assessment and the CFS in particular. One nurse reported “[The CFS] may be a useful tool but I do not know about it” while a staff surgeon reported “Not sure [the CFS] adds much beyond good history.” A majority of HCPs thought they would benefit from further training on both conducting frailty assessments in general, and in how they could use the CFS tool specifically to improve the care they provide (Table 4).

**Table 4:** Perceived need for additional frailty assessment and training score across disciplines/professions

	Nurses	Surgeons	Allied health	Total
Number of respondents	16	20	13	49
I would benefit from further training on how the CFS tool can be used to improve care in my frail patients	10/12 (83.3%) 4.3	12/16 (75.0%) 3.8	7/8 (87.5%) 4.0	29/36 (80.6%) 4.0
I would benefit from further training in how to conduct frailty assessments	12/16 (75.0%) 3.9	12/20 (60.0%) 3.6	7/11 (63.6%) 3.7	31/47 (66.0%) 3.7

Not all respondents answered all questions. Each item above represents all people who strongly agreed or agreed (numerator) divided by the number of responses to that question (denominator). The mean score (1 = “Strongly disagree”, 3 = “Neither agree nor disagree”, and 5 = “Strongly agree”) is reported below

There was no difference in the type of support between subgroups. Two-thirds of respondents identified their preferred continuing education format. Surgeons primarily described non-interactive resources (e.g. printed material, lecture) as desirable methods. Nurses and allied health professionals identified non-interactive formats as desirable but also expressed interest in interactive formats, such as group discussions and case study methods.

### Sensitivity analysis

Results from the ANOVA (Additional file 2) suggest there were overall differences in the perspectives across HCPs for the following statements: “The frailty of a patient should play a role in planning a patient’s perioperative care in the hospital” (*F*(2, 46) = 4.81, *p* = 0.013), “The frailty of a patient always plays a role in my planning of a patient’s perioperative care in the hospital” (*F*(2, 46) = 4.93, *p* = 0.012) and “Frailty is an important factor in how I provide a patient’s perioperative care in the hospital” (*F*(2, 43) = 8.75, *p* = 0.001). Specifically, the responses to “The frailty of a patient should play a role in planning a patient’s perioperative care in the hospital” were significantly different between allied health professionals and surgeons (*p* = 0.014), and between nurses and surgeons (*p* = 0.008). For “The frailty of a patient always plays a role in my planning of a patient’s perioperative care in the hospital”, responses differed significantly between allied health professionals and surgeons (*p* = 0.002). Finally, the responses to “Frailty is an important factor in how I provide a patient’s perioperative care in the hospital” were significantly different between allied health professionals and surgeons (*p* = 0.001), as well as between nurses and surgeons (*p* = 0.008). Observed differences, across the HCPs, in other survey items were not statistically significant. Both ANOVA and t-test analysis identified similar differences between each group. As we elucidated earlier, these differences of opinion were driven by surgeons being less likely to agree with these statements compared to nurses and allied health professionals.

## Discussion

Previous research has demonstrated that frailty assessment and management improve patient outcomes in both medical and surgical patients; however, it continues to have low uptake in most surgical settings. We found that hospital-based HCPs, who practice in a setting where older adult best-practices and frailty initiatives were actively underway as part of involvement in a larger EASE study, self-report that patient frailty always plays a role in the perioperative care they provide in hospital. Yet, few routinely use a frailty assessment tool to assess patients for frailty. This is especially true among surgeons, where only half of the respondents reported incorporating frailty in their perioperative care planning. A qualitative investigation by Age UK and the British Geriatrics Society revealed that frailty is viewed as something they ‘know when they see’ [28]. Numerous other studies have reported that perceived frailty varies individually and is an inadequate proxy for measured frailty [29–31]. These findings suggest that while HCPs, and surgeons in particular, acknowledge that frailty is an important factor in patients’ outcomes, most do not screen for or manage patients based on their frailty, and are unlikely to alter their care planning practices based on a patient’s frailty level. As demonstrated in the findings of this study, even among HCPs who have received education about frailty and related assessment tools, the patients’ frailty is not always factored into their care planning.

Lack of professional knowledge was self-identified as a key barrier to frailty assessments. This finding evidences both a knowledge gap and action gap. These practice gaps may also be driven by a lack of confidence in conducting frailty assessments. Indeed, only one-third of respondents, and only one-quarter of surgeons, agreed that they were confident in their ability to assess patients for frailty and the majority of respondents agreed that they would benefit from further training. The results from this survey indicated that the best approach for continuing education is a mixed approach that includes didactic teaching, group in-service sessions, and educational brochures.

A number of authors have identified similar knowledge and action gaps and have highlighted the need to close these gaps in a variety of settings [32], including frail patient undergoing planned or emergency surgery [24]. Successfully addressing these gaps will require awareness of how HCP must navigate significant system complexities and constraints in their provision of care.

Over half our respondents identified barriers to providing in-hospital care for patients who are frail. All three disciplines identified workload and staffing shortages as significant barriers; allied HCP also identified communication with the medical team and staff knowledge as barriers. These barriers may contextualize our finding that despite formalized education on frailty and the use of CFS, as a component of the EASE project, the majority of respondents (59%) were unaware of the CFS, and highlights the complexity of implementing new clinical tools. Our findings also correspond with previous research in relation to HCP-reported barriers to providing care to frail patients in acute-care settings [24, 33]. Although some participants in this study reported barriers experienced within the context of the surgical setting, more extensive investigation into HCP’s perceptions of unique barriers to providing surgical care to those who are frail is warranted.

Given that most health care delivery is based on a single problem-oriented diagnostic model, and that HCPs are not often trained to focus on the holistic care of patients, system re-organization around frailty is challenging. Furthermore, frailty is an evolving area of inquiry and consensus has not yet identified a single optimal tool to identify frailty. Without consensus on an operational definition of frailty, its practical utility is limited [34], reinforcing the knowledge-action gap. That said, the availability of validated and rapidly administered tools, such as the CFS [1], permits the use of quick, reliable and easily interpreted frailty assessments in fast-paced surgical environments. Continued education for all HCPs working in a surgical setting has the potential to improve future uptake of the CFS, and other frailty assessment instruments, towards better perioperative care for frail seniors undergoing surgery. However, given our HCPs are already in an environment examining frailty, workplace champions may be necessary to foster a culture change from the ground up.

### Limitations

The limitations of our study include a low response rate and the single-centre design. The low response rate (42%), while reasonable, somewhat limits our interpretation of the results and raises the possibility of response bias. The response rate was particularly low among surgical residents and nursing staff. This may limit the conclusions we can draw about current practices and educational programs targeting nursing and residents; that is, those who already have an interest in geriatric care may have been more likely to respond to the survey. Moreover, there were more residents (*n* = 14) than staff surgeons (*n* = 6) who responded to the survey, which resulted in fewer years of experience in a surgical setting, on average, in this group. However, when we examined the physicians and residents separately, we did not find any significant differences between their responses.

In addition to a low overall response rate, there is substantial variability in non-responses to the various survey items from the HCPs who did complete the questionnaire. In particular, 37–47% of the respondents did not provide an answer to any question in the topic of perceived usefulness of the CFS score across disciplines/professions. However, 89–96% of those non-respondents indicated they were not aware of the CFS as a frailty assessment tool. Therefore, even though our sample of responses is much lower on the topic of the CFS, those who did respond were disproportionately those who were aware of the CFS which strengthens the reliability. Overall, there was strong agreement for most items across the health professions and the results are consistent with predicted attitudes of each profession (i.e., allied health places the most importance on geriatric assessment and surgeons the least). The University of Alberta is a sizable tertiary care institution, but the single-centre design may limit generalizability to other institutions that may have more developed surgical geriatric programs.

Because the survey was conducted at a single center site, in which a larger-scale study on frailty was taking place, our findings may not be representative of other institutions in which frailty training has not been a focus. Our findings may actually reflect more positively on HCPs’ perceptions and attitudes of frailty than that of other centers. Additionally, we did not directly ask about how frailty assessment changed care pathways due to the ongoing EASE study experimental arm intervention [25], which included more frequent nursing rounds, increased physiotherapy interventions and comprehensive geriatric assessment conducted by a geriatrician for all enrolled patients.

## Conclusion

Despite the widely-recognized importance of integrating frailty and frailty assessment into perioperative care planning, little is known about HCPs’ engagement with specific tools. To our knowledge this is the first study to examine interdisciplinary HCPs’ views of frailty in a surgical setting. Our findings begin to explain HCP’s perceptions of the benefits and challenges of performing preoperative frailty assessments in an interdisciplinary surgical care setting.

The present findings highlight the knowledge-practice gaps in an institution that has introduced comprehensive geriatric assessment and frailty training. Survey respondents of the present study unanimously agreed that the frailty of a patient should play a role in planning perioperative care in the hospital. However, it is clear that the use of formal frailty assessment tools has not been widely adopted in practice and that the use and perceived usefulness of CFS, specifically, is limited. Education and further training on frailty and instruments to assess frailty may improve the integration of these tools in practice in the future. Implementation of frailty specific order sets may also improve frailty screening uptake and increase the perceived usefulness of frailty assessment. Given that a large body of literature supports the need to understand the barriers to change for optimal healthcare delivery, future implementation projects would benefit in proactively developing strategies to address the workload, communication, and knowledge barriers identified by the HCPs surveyed when introducing or implementing frailty assessments in a surgical setting.

## Additional files

Additional file 1: Survey instrument distributed to staff. (PDF 1289 kb)

Additional file 2: Pairwise comparisons of Likert scale survey responses using analysis-of-variance and post-hoc Bonferroni correction. (DOC 48 kb)

## Abbreviations

CFS: Clinical frailty scale
CGA: Comprehensive geriatric assessment
EASE: Elder-friendly approaches to the surgical environment
HCP: Healthcare professional
LPN: Licensed practical nurse
RD: Registered dietitian
RN: Registered nurse
