# Supplementary material for: Surgical frailty assessment: a missed opportunity
Source: BMC Anesthesiol. 2017 Jul 24;17:99. doi: 10.1186/s12871-017-0390-7 (PMC5525360; doi:10.1186/s12871-017-0390-7)
Supplement: Supplementary file 1 — Survey instrument distributed to staff. (PDF 1289 kb) [file 12871_2017_390_MOESM1_ESM.pdf]

UNIVERSITY OF ALBERTA

DEPARTMENT OF SURGERY
Division of General Surgery &
Division of Critical Care Medicine
FACULTY OF MEDICINE & DENTISTRY

Rachel G. Khadaroo, MD, PhD, FRCSC
Associate Professor of Surgery

2D Walter Mackenzie Health Sciences Centre
8440-112th Street
Edmonton, Alberta, Canada T6G 2B7

Tel: 780.407.7728
Fax: 780.407.7674

Invitation to participate

Study Name: The Interpret-CFS Study
Principle Investigator: Rachel Khadaroo (khadaroo@ualberta.ca)

We are conducting a survey of medical professionals working in the University of Alberta Hospital surgical wards. We are interested in your practice experience with frail patients. There is **no obligation to participate** and your employer will NOT be aware of whether you chose to participate or not. **All responses are anonymous and confidential**. No identifying information is collected, including IP address. All responses are collected using Alberta Health Services survey software. Data will be stored on secure servers in Alberta.

By completing and submitting the surveys you are providing your informed consent to participate in this study. You have the right to withdraw without prejudice or penalty until the survey is submitted. After submission, we are unable to identify your response and will be unable to remove it from the study. At all times your rights to privacy, anonymity and confidentiality will be upheld. Paper surveys will be kept for 5 years in a secure setting. Electronic survey data will be stored on secure AHS servers. Only research personnel will have access to the responses.

The study has received ethical approval from the University of Alberta REB and complies with the University of Alberta Standards for the Protection of Human Research Participants. If you have any questions about the ethical approval of this study or your rights as a research participant, you can contact the Research Ethics Office at (780) 492-2615.

Your participation in this study includes completion of **one** survey which will take no more than 5 minutes. If you have any questions or concerns regarding this study, or to request a copy of the study findings, please contact the study supervisor Dr. Rachel Khadaroo (khadaroo@ualberta.ca or 780 407 7728).

Thank you in advance for your participation.
Dr. Rachel Khadaroo

WHAT IS FRAILTY?

Frailty is defined as a clinical state of increased vulnerability due to declining health reserve and function across multiple organ systems, reducing a person's ability to cope with acute health stressors.

ABOUT THE STUDY

The Interpret-CFS study is an extension to the EASE Project, which is currently underway at the University of Alberta Hospital. Our goal is to provide insight into health care professionals' self-reported practice changes based on their interpretation and incorporation of the Clinical Frailty Scale (CFS) score in their perioperative care planning and delivery. This study will help us understand how frailty scores are used in older adults' perioperative care and provide an interdisciplinary understanding of the role of CFS in perioperative care.

DEMOGRAPHIC INFORMATION

Gender: _______

Age

- ☐ Under 25
- ☐ 25 – 34
- ☐ 35 – 44
- ☐ 45 – 54
- ☐ 55+

Discipline

- ☐ Surgeon
- ☐ Resident
- ☐ RN
- ☐ LPN
- ☐ Occupational Therapist
- ☐ Physiotherapist
- ☐ Dietitian
- ☐ Social Worker
- ☐ Other _______________

<u>Excluding interruptions (e.g., maternity leave, unemployment), how many years have you:</u>

Practiced in your current professional role: ____________

Practiced in any surgical setting: ____________

Practiced in your current surgical unit: ____________

FRAILTY: ASSESSMENT OF SURGICAL PATIENTS

Please choose a statement that reflects your beliefs about the following points (place an "X"):	Strongly agree	Agree	Neither agree nor disagree	Disagree	Strongly disagree	Don't know or not applicable
A frailty *assessment* should be done for all surgical patients						
It is part of my professional role/responsibility to assess patients for frailty						
I always use a frailty assessment *tool* to assess patients for frailty						
I am confident in my ability to assess patients for frailty						

If you use frailty assessment tool(s) in your practice, please list them here:

____

____

____

FRAILTY: MODIFYING CARE FOR FRAIL PATIENTS IN THE HOSPITAL

Please choose a statement that reflects your beliefs about the following points (place an "X"):	Strongly agree	Agree	Neither agree nor disagree	Disagree	Strongly disagree	Don't know or not applicable
The frailty of a patient should play a role in *planning a* patient's perioperative care in the hospital						
The frailty of a patient always plays a role in my *planning* of a patient's perioperative care in the hospital						
Frailty is an important factor in how I *provide* a patient's perioperative care in the hospital						
I face barriers to providing in-hospital care for patients who are frail						

Please describe any barriers you face to providing care for frail patients:

____

____

____

____

____

I am aware that the Clinical Frailty Scale (CFS) is a frailty assessment tool: Yes___ No___

CLINICAL FRAILTY SCALE SCORE						
Please choose a statement that reflects your beliefs about the following points (place an "X"):	Strongly agree	Agree	Neither agree nor disagree	Agree	Strongly agree	Don't know or not applicable
The Clinical Frailty Scale (CFS) score is useful to the overall perioperative care that is provided in the hospital						
The CFS Score is useful to the perioperative care that I provide in the hospital						
Please explain why or why not you think the CFS score is useful to your discipline:						
I would like to use or continue using the CFS score in my care of older adults						
I would benefit from further training on how the CFS tool can be used to improve care in my frail patients						

EASE STUDY INVOLVEMENT

	Yes	No
I am aware of the EASE study being conducted on ACES patients		
I am aware that the CFS is being used as part of the EASE study		
I use CFS score (posted on 3E2) for all patients enrolled in EASE to modify the care I deliver to the patient		

Please choose a statement that reflects your beliefs about the following points (place an "X"):	Strongly agree	Agree	Neither agree nor disagree	Disagree	Strongly disagree	Don't know or not applicable
Involvement with the EASE study has changed how I provide care to frail older adults						
I would benefit from further training in how to conduct frailty assessments						

What format would you prefer to receive additional training on caring for a frail patient in a surgical setting? (e.g., lecture, group discussion, printed material, mentoring)

____

____

____
