# Supplementary material for: Surgical frailty assessment: a missed opportunity
Source: BMC Anesthesiol. 2017 Jul 24;17:99. doi: 10.1186/s12871-017-0390-7 (PMC5525360; doi:10.1186/s12871-017-0390-7)
Supplement: Supplementary file 2 — Pairwise comparisons of Likert scale survey responses using analysis-of-variance and post-hoc Bonferroni correction. (DOC 48 kb) [file 12871_2017_390_MOESM2_ESM.doc]

**Additional file 2. Pairwise comparisons of Likert scale survey responses using analysis-of-variance and post-hoc Bonferroni correction**

|  | *Pairwise Comparisons* | | | | | |  |
| --- | --- | --- | --- | --- | --- | --- | --- |
|  | **Nurses vs.**  **Surgeons** | | **Nurses vs.**  **Allied health** | | **Surgeons vs.**  **Allied health** | | **Overall** |
| **Perceived importance of frailty assessment across disciplines/professions** | **Mean** | **p-value** | **Mean** | **p-value** | **Mean** | **p-value** | **p-value** |
| A frailty assessment should be done for all surgical patients | 0.29 | 0.52 | -0.23 | 0.59 | -0.52 | 0.20 | 0.45 |
| It is part of my professional role/responsibility to assess patients for frailty | 0.40 | 0.12 | 0.90 | 0.05 | 0.50 | 0.24 | 0.05 |
| I always use a frailty assessment tool to assess patients for frailty | 0.35 | 0.39 | <0.01 | 1.00 | -0.35 | 0.46 | 0.61 |
| I am confident in my ability to assess patients for frailty | 0.35 | 0.48 | 0.34 | 0.62 | -0.02 | 0.97 | 0.74 |
| The frailty of a patient should play a role in planning a patient’s perioperative care in the hospital | **0.43** | **<0.01** | 0.01 | 0.96 | **-0.42** | **0.01** | **0.01** |
| The frailty of a patient always plays a role in my planning of a patient’s perioperative care in the hospital | 0.60 | 0.07 | -0.38 | 0.29 | -0.98 | **<0.01** | **0.01** |
| Frailty is an important factor in how I provide a patient’s perioperative care in the hospital | **0.62** | **<0.01** | -0.28 | 0.23 | **-0.90** | **<0.01** | **<0.01** |
| **Perceived usefulness of the CFS score across disciplines/professions** |  |  |  |  |  |  |  |
| The CFS score is useful to the overall perioperative care that is provided in the hospital | 0.17 | 0.56 | 0.20 | 0.57 | 0.03 | 0.92 | 0.78 |
| The CFS Score is useful to the perioperative care that I provide in the hospital | 0.41 | 0.19 | 0.21 | 0.43 | -0.19 | 0.53 | 0.36 |
| I would like to use or continue using the CFS score in my care of older adults | 0.28 | 0.29 | 0.10 | 0.75 | -0.18 | 0.47 | 0.53 |
| **Perceived need for additional frailty assessment and training score across disciplines/professions** |  |  |  |  |  |  |  |
| I would benefit from further training on how the CFS tool can be used to improve care in my frail patients | 0.44 | 0.18 | 0.25 | 0.57 | -0.19 | 0.54 | 0.36 |
| I would benefit from further training in how to conduct frailty assessments | 0.34 | 0.26 | 0.21 | 0.60 | -0.13 | 0.66 | 0.52 |
